# Chain of commercialization of *Podocnemis* spp. turtles (Testudines: Podocnemididae) in the Purus River, Amazon basin, Brazil: current status and perspectives

**DOI:** 10.1186/1746-4269-10-8

**Published:** 2014-01-27

**Authors:** Jackson Pantoja-Lima, Paulo HR Aride, Adriano T de Oliveira, Daniely Félix-Silva, Juarez CB Pezzuti, George H Rebêlo

**Affiliations:** 1 Instituto Federal de Educação Ciência e Tecnologia do Amazonas (IFAM), Campus Presidente Figueiredo, Av. Onça Pintada, 1308, Galo da Serra, Presidente Figueiredo AM, CEP 69735-000, Brazil; 2 Núcleo de Altos Estudos Amazônicos (NAEA), Universidade Federal do Pará (UFPA), Campus do Guamá, Belém, PA CEP 66075-110, BRAZIL; 3 PPG- Ecologia, Laboratório de Manejo de Fauna, Instituto Nacional de Pesquisas da Amazônia (INPA), Manaus, AM CEP 69011-970, BRAZIL

**Keywords:** Chelonian, Amazon, Turtle consumption, Illegal trade, Endangered species

## Abstract

**Background:**

Consumption of turtles by natives and settlers in the Amazon and Orinoco has been widely studied in scientific communities. Accepted cultural customs and the local dietary and monetary needs need to be taken into account in conservation programs, and when implementing federal laws related to consumption and fishing methods. This study was conducted around the Purus River, a region known for the consumption and illegal trade of turtles. The objective of this study was to quantify the illegal turtle trade in Tapauá and to understand its effect on the local economy.

**Methods:**

This study was conducted in the municipality of Tapauá in the state of Amazonas, Brazil. To estimate turtle consumption, interviews were conducted over 2 consecutive years (2006 and 2007) in urban areas and isolated communities. The experimental design was randomized with respect to type of household. To study the turtle fishery and trade chain, we used snowball sampling methodology.

**Results:**

During our study period, 100% of respondents reported consuming at least three species of turtles (*Podocnemis* spp.). Our estimates indicate that about 34 tons of animals are consumed annually in Tapauá along the margins of a major fishing river in the Amazon. At least five components related to the chain of commercialization of turtles on the Purus River are identified: Indigenous Apurinã and (2) residents of bordering villages (communities); (3) of local smugglers buy and sell turtles to the community in exchange for manufactured goods, and (4) regional smugglers buy in Tapauá, Lábrea, and Beruri to sell in Manaus and Manacapuru; Finally, (5) there are professional fishermen.

**Conclusions:**

We quantify the full impact of turtle consumption and advocate the conservation of the region’s turtle populations. The Brazilian government should initiate a new turtle consumption management program which involves the opinions of consumers. With these measures the conservation of freshwater turtles in the Brazilian Amazon, is possible.

## Background

According to Alves et al. [1], at least 81 of over 700 species of reptiles in Brazil are used by, and are culturally significant to, human populations in Brazil. Of these 81 species, 30 are on the State's Red List, Brazilian Red List or International Union for Conservation of Nature (IUCN)’s Red List of Threatened Species. Lizards, snakes, caimans, tortoises, and marine and freshwater turtles are used for food, medicine, leather, ornamental and magic/religious purposes, and kept as pets [1].

The consumption of turtles by natives and settlers in the Amazon and Orinoco basins today [2,3], and also historically during the pre-Columbian period [4], has been investigated by many researchers from different scientific communities. Gilmore [4] addressed the South Americans’ and settlers’ use of animal wildlife, claiming that the poaching of Amazon river turtles was by far the most important ethnozoological activity on that continent. The capture and sale of the Giant South American Turtle (*Podocnemis expansa*) and the collection of its eggs is frequently described [1,5-7]. Egg collection is believed to have led to the extinction of this species in the upper Amazon region [7,8]. This dietary intake of meat and eggs remains clandestine, providing food and family income. Turtles are sold in regional markets despite federal prohibition legislation (law 5,197/1965) [9-14]. Several authors have noted that Amazon turtles are used in local medicine [9,15,16], and as pets and ornaments [17]. Interestingly, however, turtles are subject to well-established and highly respected food taboos. In many instances, potential consumers do avoid eating them [9]. The taboo certainly represents an important informal mechanism that could be more effective in conservation than other top-down initiatives [18,19].

Following an intense exploitation over the past two centuries, the Giant South American Turtle and the Yellow-Spotted River Turtle (*Podocnemis unifilis*) have been listed as endangered by the IUCN since 1996 [20]. These species have increased in numbers recently because of the government’s surveillance of nesting beaches [21]. Currently, *Podocnemis expansa* is listed as having a low extinction risk [22] as a result of conservation programs. The *Podocnemis unifilis* and *P. sextuberculata* are vulnerable to extinction risk [23]. Accepted cultural customs and the local dietary and monetary needs of the natives can be in conflict with conservation programs and the implementation of federal laws [18]. The adopted model of repression reduces but does not eliminate the capture and consumption of turtles in the Negro River [10,24-26], Purus River [9,27,28], and Solimões River [29].

This study was conducted around the Purus River, which is known for the consumption and illegal trade of turtles [28]. In spite of the Brazilian legislation’s declaration of illegality in 1967, the consumption of turtles in the city of Tapauá is common [30].

The majority of people consume turtles because the price is low compared with that of fish and beef [31]. Wilkie and Godoy [32] suggesting that domestic economy has changed, redefining the concept of subsistence and the patterns of consumption of bushmeat due increased of income [32]. Therefore, domestic consumers of turtles for subsistence in Tapauá are aware of such an irregularity. A variety of factors could be involved: that the consumption of turtles is accepted culturally; that the area has a population of low income [33]; and that there is an absence of state involvement, which although present in Abufari Biological Reserve (ABR), has limited performance due to the lack of human and financial resources.

Underdeveloped countries in South America have the following in common: (a) colonialism, unstable governments, and poor democracy; (b) production of items for international export including feedstock and agricultural products such as ore, wood, and other materials for natural resources–based industries; and (c) controlled by foreign investment. All of these parameters are related to the informal economy [34].

The aim of this study was to quantify the illegal trade of turtles in Tapauá and understand its impact on the local economy based on two components: (a) domestic consumption, and (b) the turtle marketing chain in the middle Purus River.

## Methods

### Study site

**Figure 1:**
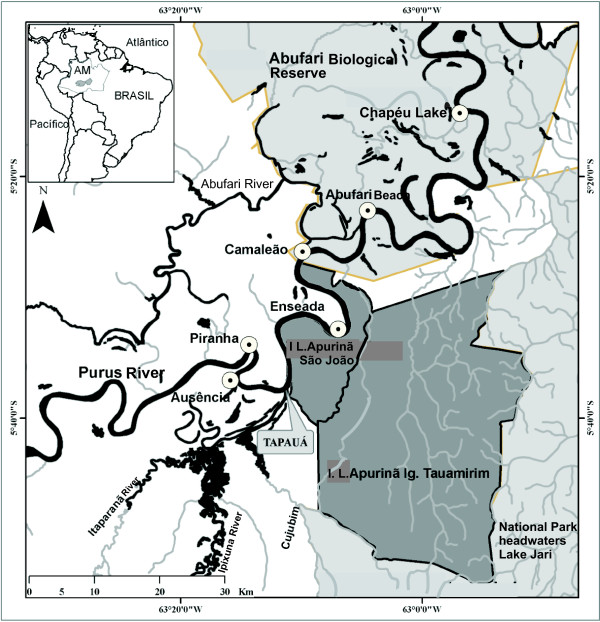
Map of the city of Tapauá (gray) in the state of Amazonas (AM) and location of fishing areas of turtles in the Purus River.

This study was conducted in the municipality of Tapauá, located 448.5 km from Manaus, capital of the state of Amazonas (05°37′S and 63°11′W) (Figure 1). Tapauá has an estimated population of 20,000 inhabitants and an urbanization rate of 55.66% in 2000 census [33]. In 2007 Tapauá had 4,080 private households, of which 3,704 were inhabited [33]. About 68% of the population is low income, based on the Brazilian Institute of Geography and Statistics (IBGE)’s 2000 census [33]. Inhabitants are mostly descendants of migrants from the miscegenation of the Brazilian Northeast and indigenous ethnicities (such as Apurinã, Palmari, Jamamadi, and Catauaxi [30,35]). The city of Tapauá was established in 1938 as an administrative district of the city of Canutama and declared a municipality in 1955 [30].

### Procedures

To estimate turtle consumption, interviews were conducted over 2 consecutive years (2006 and 2007) during the summer period, which is the high-consumption season in the Amazon (July to December in previous years). The interviews were conducted in urban areas and isolated communities. In January of 2006, 101 interviews were conducted (72 in urban areas and 29 in rural areas). In 2007, 124 interviews were conducted in urban areas only due logistics conditions.

The experimental design was randomized with respect to households. Residents interviewed belonged to all age groups (14–80 years) and differed only in schooling (incomplete primary [55%] or functionally illiterate [24%]; formal education completed: high school [29%], primary education [25%], and incomplete high school [24%]). Data on age were grouped into five age classes (Class I: 14–20, Class II: 21–30, Class III: 31–40, Class IV: 41–50, and Class V: over 50 years). In January of 2007, the consumption of 2006 was classified by classes of annual consumption per household (Class I: 1–5 animals/year; Class II: 6–10 animals/year, Class III: 11–15 animals/year; Class IV: 16–20 animals/year, Class V: 21–25 animals/year; Class VI: over 25 animals/year). In addition, the following was obtained: frequency of egg consumption; perception of environmental legislation; the number of animals consumed per household, per species; and the purchase price and origin of animals consumed.

Consumption data collected in 2006 were analyzed according to the measure of central tendency for grouped data [36]. The Student *t*-test was used to compare total consumption of turtles among households in rural and urban areas (2006 data). A G-test was used to compare consumption frequency within the consumer classes (I–VI), between the areas of the municipality (urban and rural), age classes (I and V), and the education level of the respondents.

Continuous data consumption and purchase price obtained in December 2007 were explored using descriptive statistics [36]. From the 2007 analysis were produced estimates of total consumption (EC), biomass (B), consumption per capita (CP), estimated expenditure (EE), and expenditure per capita (EP) as shown in the equations below:

(1) EC=Fr.∗2356∗C

where EC is the estimated number of animals consumed; Fr. is the percentage of households (0–1) where the consumption of the species occurred (2,356 = number of occupied households in the urban area [37]; C is the median intake of animals by species in each household because the values of consumption, purchase price, and expenses do not meet normality [36].

(2) B=Fr.∗2,356∗EC

(3) $$\mathrm{CP}=B/\left(\mathrm{Fr}.*10,013\right)*\mathrm{kg}$$

where B is the estimated biomass of animals consumed in the urban area of Tapauá; CP is the per capita consumption in kilograms (10,013 total population of the urban area of Tapauá in 2007 [37]); kg is the average weight of turtles (*P. sextuberculata* = 0.9 kg, *P. unifilis* = 2.88 kg; *P. expansa* = 4.99 kg). The average weights were calculated from animals seized during surveillance of the ABR by the Brazilian Institute of Environment and Renewable Natural Resources (IBAMA) [28] and from experimental animals caught in the fishery in 2007.

(4) EE=EM∗Fr.∗2356

where EE is the estimated expenditure for the purchase of real turtles in Tapauá; EM is median spending per household in the urban area of Tapauá.

(5) $$\mathrm{EP}=\mathrm{EE}/\left(\mathrm{Fr}.*2356\right)$$

where EP is per capita spending in U.S. dollars. Median values estimated by Equations 1–5 are given a percentile followed by the 25% and 75% obtained, which corresponds to the original data.

To study the turtle fishery and trade chain we used snowball sampling methodology [38], with the first key informant (“who makes a living catching turtles and could give an interview?”) indicated by the community. Interviews were conducted in January 2007 with four key interviewers in Tapauá, mainly participants of turtle artisan fishermen groups. Information such as the number of fishing events per year, number of days using a fishery, number of people involved in fishing, number of teams working on fishing, teams’ average number of weekly fishing events by week, different kinds of fishing artifacts (dimensions and mesh size), and yield (number of turtles caught by species, sex of animal, estimated size, the amount of sales, and gross changes in prices in Brazilian currency) was estimated.

We developed a model of the supply chain with key components identified in the following categories: (a) free-narrative interviews with three regional fishermen on boats in the Purus River lines with cargo of a capacity between 50 and 100 tons, (b) interviews with four turtle artisan fishermen, (c) interviews with 196 residents of urban areas and 29 of rural areas, (d) the recorded seizures [27,28], (e) research in the Abufari reproductive area; (f) study of the resource use and turtle ecology in the Abufari area, and (g) experimental fisheries. From this model we constructed four turtle conservation scenarios in the Purus River floodplain.

## Results and discussion

**Figure 2:**
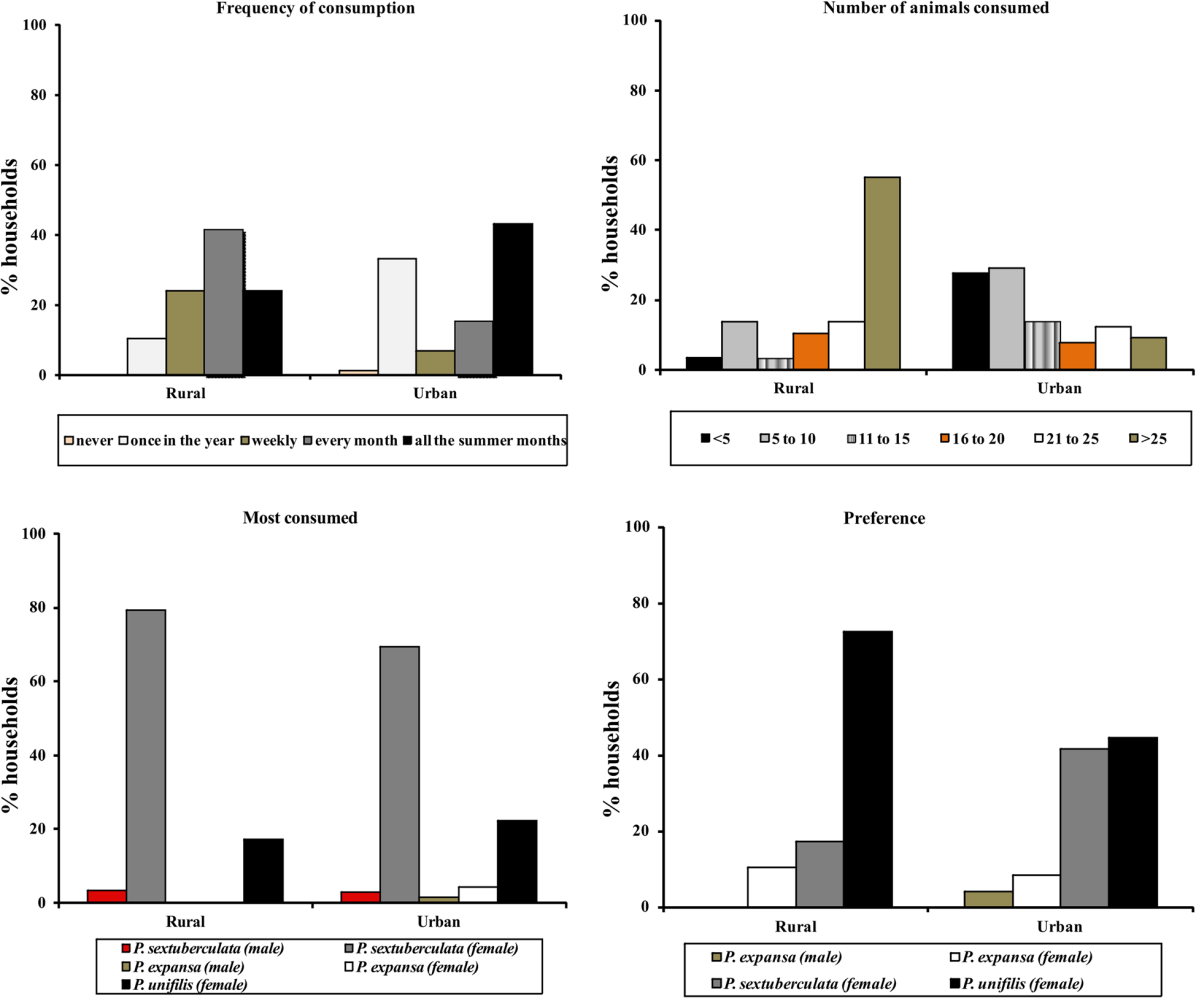
Results of interviews on the consumption of turtles in the city of Tapauá, Amazon, Brazil in 2006.

Consumption of turtles occurred in 100% of urban and rural households (a total of 101) in 2006. A study by Rebêlo and Pezzuti [12] showed that in the city of Novo Airão, 18.8% of interviewees reported never having consumed turtles. In Manaus these indices were higher among suburbs (44.4%) and University of Amazonas students (58.3%) [12]. In Tapauá, urban consumption occurs through all months of the year (41.4% of households in the city), while in rural areas it occurs mainly during the summer (July–December in 43.1% of households [Figure 2]).

In rural areas the Yellow-Spotted River Turtle (*P. unifilis*) (72%) is the preferred species, while in the city preferences were split between the Six-tubercled River Turtle (*P. sextuberculata*) (41.7%) and the Yellow-Spotted River Turtle (44.5%), species of small and medium size, respectively. Rebêlo and Pezzuti [12] have shown that *P. unifilis* and *P. expansa* were the most preferred among interviewers of Manaus, Novo Airão, and Jaú National Park, perhaps due to the size of these species in relation to *P. sextuberculata*[12]. The most consumed species in urban and rural areas of Tapauá was *P. sextuberculata* (Figure 2). The Yellow-Spotted River Turtle was actively consumed in all sites evaluated by Rebêlo and Pezzuti [12], but the most consumed in Jaú National Park was the Big-headed Amazon River Turtle (*Peltocephalus dumerilianus*) in all years of the consumption monitoring [9,10,12,24,25]. Most chelonians marketed in Tapauá weighed less than 2 kg, similar to those described at Itacoatiara [8] and Jaú River [12].

Egg consumption occurred only in the summer, when all species perform their nesting. Eggs consumed were mostly from the Six-tubercled River Turtle (*P. sextuberculata*), although the preferred eggs were from the Yellow-Spotted River Turtle (*P. unifilis*). In households of Tapauá, the assessed consumption was an average 14.57 ± 9.56 kg of turtles per household for six months of the year (July to December). The average turtle consumption was greater in rural areas (21.65 ± 7.99 turtles per household) than in urban (11.26 ± 8.27 turtles per household) (*t* = -5.767, GL = 99, p < 0.001) in 2007. This pattern was also observed for grouped dates collected in 2006, showing that a greater consumption of turtles appears more in rural than in urban areas (GWillians = 27.449, GL = 5, p < 0.001). In urban areas consumption predominates in Classes I (27.7%), II (29.2%), and III (13.8%), while in rural households consumption occurs most frequently in Classes II (13.8%), IV (13.8%), and VI (55.2%). There was no significant difference in consumption frequency regarding the age of respondent (GWillians = 22.267, GL = 20, p = 0.326). It was found that the education level influences the frequency of consumption per household (GWillians = 46.351, GL = 88, p = 0.0007). Respondents with incomplete primary education account for 19.4% of consumers in Class V (7.5%) and VI (11.8%), while respondents with high school education, complete and incomplete, account for 18.3% of the recorded consumption frequency of Classes I and II.

The origin of turtles consumed in the city (66.7% of households) and in rural areas (55.2% of households) was not specified. When asked if animals captured in the ABR were consumed, 86.2% of households in rural areas and 88.7% of urban areas said yes, which corroborates the observations of Ferrarini [30]. Most of the respondents in urban (79.4%) and rural areas (58.0%) did not agree with the law that completely prohibits trade instead of defining what is and is not allowed. The interviewers recognized captive breeding (54.2%) and management (38.9%) as the best solutions among the options discussed (no opinion: 18.1%; do not eat more turtles: 16.7%; no restriction for capture in the wild: 12.5%).

In all of the 124 selected households evaluated in 2007, at least one chelonian was consumed every year (Table 1). In terms of the most-consumed species the results are similar to the 2006 interviews, in which the Six-tubercled River Turtle (*P. sextuberculata*) was the most consumed. Financially, the Giant South American Turtle (*P. expansa*) has a higher market value (Table 2). The maximum spending per household was $219.29 U.S. dollars a year for three species, which shows consumption at all economic levels.

**Table 1:** Number and percentage of households consuming freshwater turtles in the city of Tapauá in 2007

**Species**	**N(%)**	**Animals**	**C**	**EC**	**B**	**CP**
*P. sextuberculata*	98 (79)	1,102	8 (5–15)	14,888 (9,305–27,915)	13,399 (8,374–25,123)	7.2 (4.5–13.5)
*P. unifilis*	69 (55)	169	2 (1–3)	2,620 (1,310–3,930)	7,545 (3,773–11,318)	5.76 (2.88–8.64)
*P. expansa*	74 (59)	198	2 (1–4)	2,812 (1,406–5,624)	14,032 (7,016–28,064)	9.98 (4.99–19.96)

N (%) = number of animals whose consumption was reported; C = mean consumption per household; EC = number of animals estimated; B = estimated biomass consumed (in kg); CP = Consumption per capita (g/day). Values brackets represent 25% and 75% percentiles.

**Table 2:** Median spending per household as declared in January 2007 and estimated annual expenses with the purchase of turtles by species in urban Tapauá

**Species**	**Average spending by household (US$)**	**Annual estimated spending (US$)**
*P. sextuberculata*	13.05 (7.99–23.19)	24,307.62 (14,888.26–43,176.16)
*P. unifilis*	28.72 (16.65–45.96)	37,651.18 (21,822.52–60,241.89)
*P. expansa*	80.10 (36.97–147.41)	112,625.33 (51,980.72–207,238.82)

Values brackets represent 25% and 75% percentiles.

In 2006, over 34 tons (living biomass) of turtles were acquired by consumers. Of this amount, 40.1% was *P. expansa*, 38.3% was *P. sextuberculata*, and 21.6% was *P. unifilis*, for food consumption in Tapauá (Table 1). This consumption per capita was 15.9 g/person/day. The average expenditure was estimated about $200 U.S. dollars in the summer of 2006 (Table 2) in the city of Tapauá only. The estimated consumer spending in Tapauá was $175,000 U.S. dollars, representing 2.71% of resource transfers from the Brazilian Federal Government in 2007 ($6,443,206.08 U.S. dollars) for this city [39]. Tapauá is a city with a low Human Development Index and great social inequality, as measured by the Gini Index [33], suggesting that the consumption of smaller species is directly related to lower social status. The population with a higher purchasing power acquires more valuable species, such as the Giant South American Turtle (*P. expansa*), which can cost seven times more than the Six-tubercled River Turtle (*P. sextuberculata*) and three times more than the Yellow-Spotted River Turtle (*P. unifilis*).

The turtle fisheries last about two and a half days and gather groups of up to 6 anglers. It was estimated that there were 20 groups who sell a production in the municipality (between 45 and 100 fishermen). The turtle artisan fishermen use modern techniques, known locally as “capasaco,” to increase fishing yields but these methods also lead to a high proportion of damaged turtles that cannot be sold.

We identified six main fishing spots (Figure 1). Three were within the limits of ABR and three were in neighboring areas. In 16 weeks (August–November), each fisherman profited an average of $2,300 U.S. dollars ($175.00 U.S. dollars/fishermen/week).

**Figure 3:**
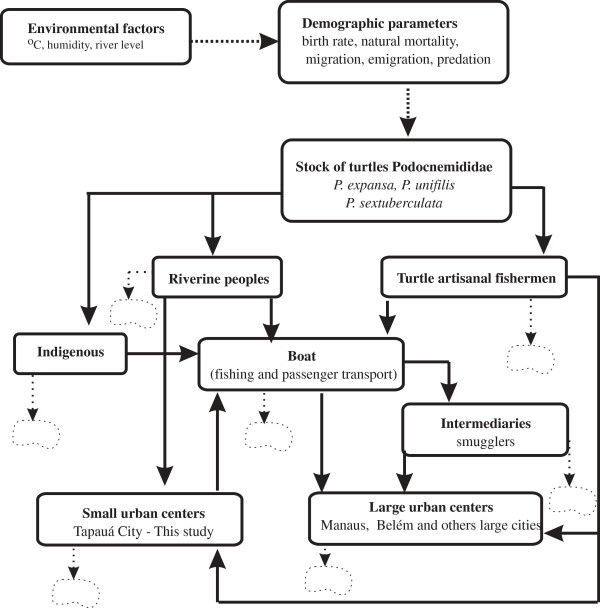
**Compartment model of chain marketing of turtles in the Purus River.** Boxes indicate the social actors of the chain; arrows indicate the direction in which the resource is being conducted; open clouds indicate indeterminate destination resource or raw.

The components of the commercial chain (Figure 3) are (1) indigenous Apurinã and (2) residents of bordering villages (communities), both of which capture and collect turtle eggs mainly for food (subsistence). Another group (3) of local smugglers buy and sell turtles to the community in exchange for manufactured goods, and (4) regional smugglers buy in Tapauá, Lábrea, and Beruri to sell in Manaus and Manacapuru. These traders use intermediaries who resell at higher prices. Finally, (5) there are professional fishermen who have mastered the catching techniques and invest time and money during 4 months (August to November) solely to capture turtles.

In the Purus River area, 100% of respondents in the 2 years of monitoring reported consuming at least three species of turtles (*Podocnemis* spp.). From July to December, extensive sandbars arise in the Purus River, which turtles use for nesting [25]. Researchers such as Wilkie and Godoy [32] estimate that an increase in income leads to a reduced consumption of game meat, but the present study refutes this theory. Several studies have shown that consumption and commercialization of turtles in Amazonia is a habit rooted in the culture of local peoples [8-10,12-14]. The present study shows that the age of respondents did not influence the frequency of consumption, corroborating the idea that consumption in Amazonia is cultural. In the state of Amazonas, people consume turtles weekly, as seen in Novo Airão, while in Manaus consumption is less frequent [12].

In the city of Tapauá weekly consumption of turtles is more common among respondents in rural areas, especially during the summer. Fish is the main source of animal protein for Amazonian riverside populations, and per capita consumption in the Amazon between different areas varied from 165 g/person/day or 60.0 kg/person/year in Monte Alegre [40], to 500–800 g/person/day or 182.5–292 kg/person/year on the Solimões River [41]. Besides the consumption of fish, game animals were measured at a per capita consumption of 13.6 g/person/day in the middle Amazon [40]. In Tapauá, per capita consumption of turtles is higher (15.9 g/person/day of turtle), but this value reflects the biomass of live animals. The yield of *P. expansa* ranges from 20.7% [42] to 50% of the weight of turtles without the hull [43].

The consumption of turtles has other nutritional benefits as well as being a good source of protein and have one specific market mainly *P. expansa* and *P. unifilis* that are preferred by many people [7,26,44,45]. The hull of *P. expansa* is rich in calcium and phosphorus, and contains significant amounts of iron, zinc, copper, manganese, and cobalt [47]. A study performed in the region of Pracuúba (Amapá State, Brazil) shows that of the 35 plant species that are part of the turtles’ diet, 12 present protein greater than 10.0%, 4 have lipid content higher than 10.0%, 9 have high content of crude fiber, 6 have more than 5.0% of mineral matter, 6 have more than 1.0% calcium, and 5 have more than 0.2% phosphorus [48]. In captivity, the meat from the males of *P. expansa* has higher levels of copper, calcium, and phosphorus, while the meat of the females has higher sodium and magnesium [49]. The meat of the Giant South American Turtle (*P. expansa*) has higher levels of calcium (mean 189 mg to 242 mg for females and males, respectively) to those found in beef (7 mg) and chicken (12 mg) [50,51]. From an environmental standpoint, turtle consumption has a heavy impact because it removes long-living organisms that are responsible for processing energy, nutrient cycling, dispersal of riparian vegetation and maintenance of water quality in the lowland ecosystem [46].

Our estimates demonstrate that thousands of animals are consumed annually in Tapauá along the margins of a major fishing river in the Amazon [52,53] where fish is the main food resource. However, it is unclear what impact this activity has on natural turtle populations. It can be observed that the trading price of the Six-tubercled River Turtle (*P. sextuberculata*) is lower than the other two species (Table 1), equating to the price of chicken meat per kilo ($3.50 U.S. dollars). The purchase price of the three species of turtle is less than beef on average in Tapauá ($4.40 U.S. dollars/kg, personal observation).

The capture and trade of turtles in Tapauá generates income for fishermen, but its illegality excludes it from official tax statistics. The gross domestic product (GDP) of the city of Tapauá in 2005 was approximately $40,000 U.S. dollars, from the provision of services and agricultural activities [33]. The activity is profitable, but has a risk of fines and seizure by environmental protection agencies. Although the IBGE names fishing as the largest source of employment and income generation in Tapauá, the turtle artisan fishermen interviewed did not have a high social status and were eligible for government welfare benefits [33].

Concerned with the need for turtle conservation in the Purus River, respondents identified the development of captivity and domestication as the main alternative to the present situation. For human ecology, environmental policies seek to change the habits of the population. Although both rural and urban consumers refer to the idea of conservation reform, only a small portion of respondents in 2006 (1%) and none of those 2007 have never eaten turtle. Among the options proposed by respondents, one alternative is a more coherent quota management of the wild areas, similar to “participatory management” proposals [12,24,25] in which users manage the natural resources. Caputo et al. [54] show that nest community management is efficient and can be done at low cost. The second alternative was making captive breeding more viable. Podocnemidids grow slowly, and consume 1.2–1.5 grams of fish feed per day [55]. It has the problem of a low-income hull [44,45] which increases the cost and market price.

Consumption management can involve the community and the suppliers of young turtles taken from nature, but the federal laws 5197/1967 and 9605/1998 (regulatory frameworks of environmental management in Brazil) do not make this possible. We considered that all stakeholders of resource turtles in the lower Purus River should be involved in an integrated process, as has occurred in other regions of the Amazon basin [24,29,54]. Integration is imperative because animal use and exploitation, combined with the cultural aspects of human interaction with animals, can contribute to pressure on animal species, leading to either their sustainable use or extinction [56].

Several authors posit that habitat destruction and predatory use was the main threat to the natural populations of reptiles [13,26,46]. Alves and Santana [13] state that it is essential for conservation and management programs to involve the local communities who exploit the natural resources. Community-based efforts are limited by scarce funding, consistent and effective involvement of stakeholders, and political infighting [13]. Conway-Gomez [57] argues that a management strategy has the most potential to redirect human behavior from unrestrained exploitation to the sustainable use of a resource.

Other authors recommend community-managed captive breeding of the faster-maturing *P. unifilis* and *P. vogli* in the Orinoco Basin to satisfy turtle consumption needs. These measures, along with improved nesting-beach protection, may encourage the recovery of populations of *P. expansa* and make their legal subsistence harvesting possible in the future [58]. These authors recognize that “*after 21 years of protecting turtles in and around the Arrau Turtle Wildlife Refuge (AWR), it has become obvious that using force to eliminate consumption of this traditional staple is not an option in the Middle Orinoco. The consumption of* P. expansa*,* P. unifilis*, and* P. vogli *are deeply rooted in the lifestyle and economic reality of the ribereño*” [58].

It may be that catches are sustainable, and long-term monitoring will be able to determine this sustainability. Managing these resources through participatory planning and an integrated ecosystem-based plan is not currently possible because the law prohibits all turtle use.

## Conclusion

Our results corroborated that consumption of *Podocnemis* spp. turtles is common in the Amazon Basin, particularly along the Purus River, where the major nesting site of turtles is located in the state of Amazonas.

We believe that our results evaluate the full impact of turtle consumption and advocate the management of turtle consumption to contribute to the conservation of the region’s turtle populations. Our data show that consumption occurred independent of age and social class. Thus, it is clear that the Brazilian government should alter the paradigms currently in place and initiate a new turtle consumption management program that includes users in decision making and would indeed contribute to the management and conservation of freshwater turtles in Brazil, particularly in the Brazilian Amazon.

## Competing interests

The authors declare that they have no competing interests.

## Authors’ contributions

JPL, JCBP, DFS, and GHR conceived the study, and participated in its design and coordination. JPL collected the dates during two years on the city of Tapauá. PHRA and ATO analyzed the data and drafted the results for the discussion. All authors helped to draft the manuscript and read and approve the final manuscript.
